# 517. Safety and Tolerability of Intramuscular (IM) Sotrovimab 500 mg Administered at Different Injection Sites: Results from the Phase I COSMIC Study

**DOI:** 10.1093/ofid/ofad500.586

**Published:** 2023-11-27

**Authors:** Jennifer Moore, Wen-Hung Chen, Jerzy Daniluk, Sergio Parra, Megan Turner, Yasmin Sanchez-Pearson, Prosenjit Sakar, Ian A Hawes, Alicia Aylott

**Affiliations:** GlaxoSmithKline, Brentford, England, United Kingdom; GSK, Collegeville, Pennsylvania; GSK, Collegeville, Pennsylvania; Vir Biotechnology, San Francisco, California; GSK, Collegeville, Pennsylvania; GSK, Collegeville, Pennsylvania; GSK, Collegeville, Pennsylvania; GSK, Collegeville, Pennsylvania; GSK, Collegeville, Pennsylvania

## Abstract

**Background:**

Sotrovimab is a dual-action Fc-engineered human monoclonal antibody (mAb), developed for early treatment of mild-to-moderate COVID-19. We evaluated the safety and tolerability of sotrovimab 500 mg administered intramuscularly (IM) at dorsogluteal (DG), anterolateral (AL) thigh and deltoid injection sites.

**Methods:**

This Phase 1 open-label, parallel group, randomized, healthy volunteer study comprised three parts. Part A investigated the relative bioavailability, safety and tolerability of 2 sotrovimab concentrations administered IM at different injection sites. 216 participants were randomized (2:2:1:1) to 4 treatment arms: 62.5 mg/mL DG administered as two 4 mL injections; 100 mg/mL DG administered as one 5 mL injection; 100 mg/mL AL thigh administered as one 5 mL injection; 100 mg/mL deltoid administered as one 2.5 mL injection into each muscle. Incidence of adverse events (AEs), serious AEs (SAEs) and AEs of special interest (AESI) through Day (D) 29 were assessed. Patient-reported outcomes included Perception of Injection (PINS) v3 and Pain-Numeric Rating Scale (NRS).

**Results:**

215 participants were exposed to study intervention (mean [range] age: 39 [19–65] years; 57% female; mean [standard deviation] body mass index: 25.4 [2.87] kg/m^2^). Overall, 46% (n=99/215) of participants reported AEs; most common ( > 5%) were injection-site pain (30%, n=65/215) and COVID-19 (6%, n=12/215; study day of diagnosis: range 1–97 days, median 43 days). There were no SAEs; all AESIs were Grade 1 or 2 (Table 1). Mean Pain-NRS values were < 1 and similar across injection sites; values were highest on D1 at 15 mins post-dosing and then decreased through 60 mins post-dosing (Figure), with 8 participants reporting pain after D2. D1 PINS results showed the majority felt injection site reactions were “not at all” bothersome, and local reactions and pain were “totally or very acceptable”. No or little impact on sleep and movement were reported (Table 2).

**Table 1.**
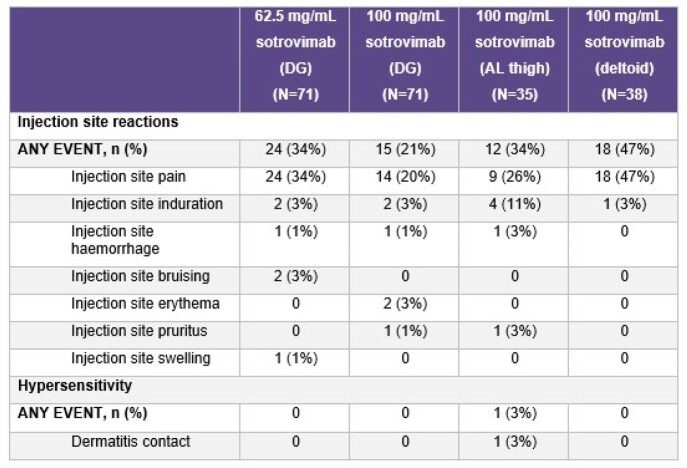
AESIs AESI, adverse event of special interest; AL, anterolateral; DG, dorsogluteal

**Table 2.**
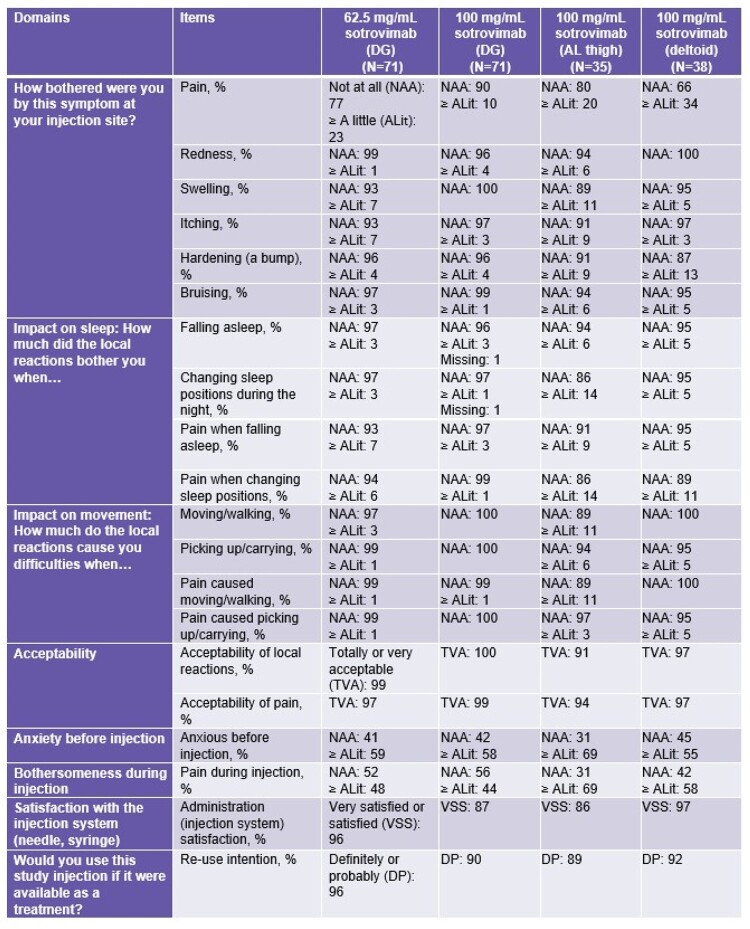
PINS results (Day 1) AL, anterolateral; ALit, A little; DG, dorsogluteal; DP, definitely or probably; NAA, not at all; PINS, Perception of Injection; TVA, totally or very acceptable; VSS, very satisfied or satisfied

**Figure.**
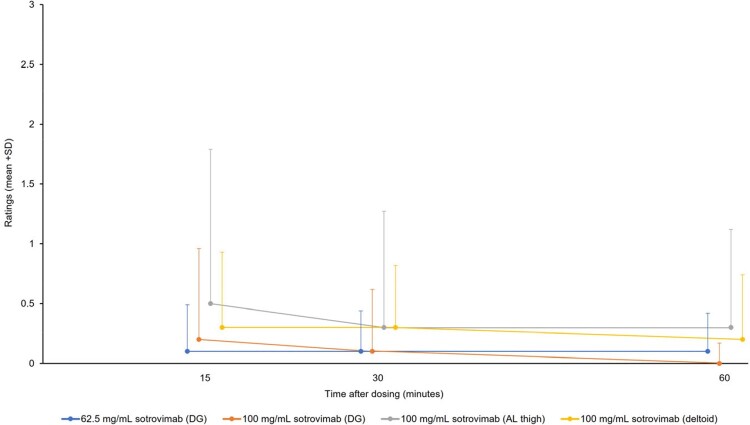
Mean (+SD) Pain-NRS over time by treatment arm (Day 1) Pain-NRS is scored from 0–10; Figure only shows 0–3 to represent the observed summary statistics from Day 1. AL, anterolateral; DG, dorsogluteal; NRS, Numeric Rating Scale; SD, standard deviation

**Conclusion:**

Sotrovimab 500 mg IM injection had a favorable safety profile and was generally well tolerated at all injection sites, and at volumes of up to 5 mL at DG and AL thigh sites, and one 2.5 mL injection into each deltoid muscle. To our knowledge, these are the first data to show that a 2.5 mL mAb injection into each deltoid muscle was well tolerated.

**Disclosures:**

**Jennifer Moore, MD**, GSK: Employee|GSK: Stocks/Bonds **Wen-Hung Chen, PhD**, GSK: Employee|GSK: Stocks/Bonds **Jerzy Daniluk, MD, PhD**, GSK: Employee|GSK: Stocks/Bonds **Sergio Parra, MD**, Vir Biotechnology, Inc: Employee|Vir Biotechnology, Inc: Stocks/Bonds **Megan Turner, BA**, GSK: Employee|GSK: Stocks/Bonds **Yasmin Sanchez-Pearson, PhD**, GSK: Employee|GSK: Stocks/Bonds **Prosenjit Sakar, MSc**, GSK: Employee|GSK: Stocks/Bonds **Ian A. Hawes, BSP, ACPR**, GSK: Employee|GSK: Stocks/Bonds **Alicia Aylott, MSc**, GSK: Employee|GSK: Stocks/Bonds

